# Between Lived Experience and Professionalisation: Can Personal Assistance Redefine Peer Support in Mental Health?

**DOI:** 10.3390/healthcare14030346

**Published:** 2026-01-29

**Authors:** Javier Morales-Ortiz, Francisco José Eiroa-Orosa, Juan José López-García, Mª Dolores Pereñíguez

**Affiliations:** 1 Faculty of Psychology and Speech Therapy, University of Murcia, Espinardo University Campus, Building No. 31, 30100 Espinardo, Murcia, Spain; j.moralesortiz@um.es (J.M.-O.); jjlopga@um.es (J.J.L.-G.); 2 AFESMO—Association for Mental Health Molina and Its Region, Gregorio Miñano, 52, 30500 Molina de Segura, Murcia, Spain; 3 Section of Personality, Evaluation and Psychological Treatment, Department of Clinical Psychology and Psychobiology, School of Psychology, Institute of Neurosciences, University of Barcelona, Passeig de la Vall d’Hebron, 171, 08035 Barcelona, Spain; feiroa@ub.edu; 4 Faculty of Nursing, Catholic University “San Antonio” of Murcia, Los Jerónimos Campus, Av. de los Jerónimos, 135, 30107 Guadalupe, Murcia, Spain

**Keywords:** peer support, lived experience workforce, personal assistance, mental health, professional identity

## Abstract

Background/Objectives: The incorporation of peer support within mental health services has shown benefits for service users’ recovery and engagement, yet implementation is often hindered by role ambiguity and limited institutional recognition. The aim of this study is to explore the experiences of workers in a programme that provides peer support within a personal assistance model. The focus is on how they perceive the shaping of their professional role and their integration within care teams, rather than on evaluating service outcomes or effectiveness. Methods: An interpretive qualitative methodology with an exploratory approach was used. The study was conducted in a single organisational setting and focused on the self-reported experiences of personal assistants. Fieldwork was conducted in 2025 with ten personal assistants. Data were obtained through individual semi-structured interviews and one focus group with the same participants. A thematic content analysis combining inductive and deductive coding strategies was conducted using MAXQDA (version 24.11). Results: Findings indicate that the Personal Assistant role was perceived as reducing some of the ambiguity commonly associated with peer support, due to a clearer contractual framework and a more explicit delineation of functions. However, tensions persisted in relation to its hybrid professional identity, experiences of task overload, and ongoing gaps in coordination with traditional professional roles. Key facilitators included institutional support, accessible coordination, a supportive culture of care, and informal peer networks. Perceived benefits were reported for service users, including increased trust, hope, and autonomy, as well as for assistants, who described enhanced professional purpose and progress in their own recovery, alongside risks of emotional strain. Conclusions: Analysing the perspective of participants, the personal assistance model may represent a promising framework for the professionalisation of peer support through functional clarity, continuous supervision, and recognition of experiential knowledge. Further progress requires strengthening internal communication, expanding training opportunities, and enhancing the structural participation of personal assistants in decision-making. The study contributes an exploratory qualitative perspective to the growing literature on integrating lived-experience professionals into mental health services.

## 1. Introduction

In recent years, mental health services have evolved toward models grounded in recovery and the protection of users’ rights [1]. Within this framework, peer support has emerged as a practice that complements traditional care and provides well-established benefits for both service users and those who perform this role [2,3,4].

Peer support workers draw on their lived experience to provide support grounded in empathy and mutuality, fostering hope, self-efficacy, and engagement with services or adherence to therapeutic recommendations [4]. However, the literature has consistently identified the lack of a clearly defined role as a central challenge in the implementation of these programmes [5]. This ambiguity, described by some authors as the notion of liminality [6], can generate confusion within teams, overlaps in responsibilities, and even tensions concerning the boundaries between the personal and the professional [7].

Professional identity is shaped through the interplay of personal history, work experience, and a sense of belonging to a group, all within a social and organisational context that evolves over time [8]. It is therefore expected that peer support workers construct their professional identity at the intersection between lived experience and increasing professionalisation, a process that has been associated with role tensions and identity ambiguities [5]. Establishing a clear definition of the role in terms of functions and tasks, as well as clearly articulating explicit relational boundaries, emerges as a key element in the effective implementation of these programmes [9].

A growing body of research indicates that the degree to which peer support workers are integrated within mental health services depends on organisational climate and the presence of clear support structures [10,11], as well as the availability of specific training programmes [12,13]. Recent studies [14,15] suggest that the institutional context plays a decisive role in the success of peer support initiatives. In Spain, despite increasing interest in incorporating peer support workers into mental health teams, their inclusion remains limited and uneven. This is largely hindered by challenges related to employment pathways for peers and the institutional recognition of the professional role [16].

In Spain, the integration of peer support within mental health services is shaped by specific legislative and social service frameworks that provide a unique context for this study. Law 39/2006 on the Promotion of Personal Autonomy and Care for Persons in Situations of Dependency has represented a significant step forward in shaping the portfolio of social services. It has expanded the resources available to people with disabilities and facilitated access to personalised supports aimed at autonomy and community inclusion. Within this regulatory framework, personal assistance is defined as an individualised support service that enables users to exercise greater control over their daily lives by assisting with basic and instrumental activities, social participation, access to education or employment, and decision-making related to their life project. Specifically, the tasks that personal assistants may undertake include support with basic daily activities, assistance in organising the home environment, help with mobility and transportation, accompaniment to medical appointments, leisure activities or community engagement, and support with administrative procedures, paperwork, and appointments. Although its application within the field of mental health remains limited [17], several initiatives, such as those developed in the region of Castilla y León, have demonstrated its potential to enhance social participation while also offering an appropriate employment framework for peer support and the professional integration of individuals with lived experience [18].

In this context, in the city of Molina de Segura (Murcia, Spain), the Association for Mental Health Molina and its Region (hereafter AFESMO by its acronym) has developed an innovative approach that integrates peer support within the formal framework of personal assistance. This model not only establishes a clear structure for the employment relationship and the functions associated with the role but also contributes to strengthening the professional identity of peer supporters, both in relation to service users and within teams [19].

To our knowledge, this is the first study to examine the experiences of personal assistants who deliver peer support within a formally regulated personal assistance framework. As a pioneering programme in the Spanish context, this model offers a unique opportunity to explore how embedding peer support within the long-term care system may influence role clarity, professional identity, and integration within mental health teams. Accordingly, this study aims to explore how personal assistants perceive and experience their work within the personal assistance model, to examine how this framework shapes their professional identity in comparison with other peer support approaches, and to identify key elements that may contribute to the implementation of peer-based personal assistance programmes. Given its exploratory nature, the study provides an initial interpretative contribution and points to the need for further research with larger samples and comparative designs.

## 2. Materials and Methods

### 2.1. Study Design

A qualitative methodology was employed to gain an in-depth understanding of the experiences, meanings, and perceptions [20] of personal assistants with lived experience in mental health, particularly concerning their professional role and their integration within care teams. This methodological orientation is grounded in the premise that reality is best understood from the perspective of those who live it, emphasising the subjective meanings individuals assign to their own experiences. Following the perspective proposed by Taylor and Bogdan [21], who argue that qualitative research involves engaging with individuals to understand reality from their own frame of reference, our methodological orientation was grounded in the need to give voice to the participants and to understand the phenomenon through their lived experience.

Two complementary data collection techniques were employed: semi-structured interviews and a focus group. The combined use of both methods allowed for access to different levels of understanding of the phenomenon under study. On the one hand, following Vallés [22], individual interviews enabled an in-depth exploration of personal experiences, biographical nuances, and the meanings each personal assistant attributed to their role as a peer support agent. On the other hand, the focus group provided an opportunity to observe how these experiences were shared, negotiated, and contrasted through interaction with others, thereby capturing the collective and relational dimensions of the discourse [23,24]. The complementarity between both techniques enriched the analysis, offering a broader and more contextualized understanding of how personal assistants with lived experience in mental health construct their professional role.

### 2.2. Description of the Personal Assistance Programme

The Personal Assistance programme is implemented within a community-based mental health organisation and integrates peer support within a formally regulated assistance framework. All personal assistants are required to hold formal training in socio-health care for dependent persons and to have lived experience of mental health problems. Recruitment and selection are conducted internally by the organisation’s management team. In addition to the qualifying socio-health care training, all assistants complete an online course focused on Personal Assistance and receive internal training on the organisation, its resources, and the specific service in which they work. Personal assistants are supported through daily coordination and supervision provided by a designated coordinator, who accompanies practice, supervises interventions, resolves difficulties, and organises work schedules. Assistants also have access to a psychologist within the organisation if psychological support is needed. Supervision is primarily organisational and supportive rather than clinical. Workload is adjusted to contractual working hours, with the organisation prioritising full-time or more than half-time contracts to support stable and sufficient income. Personal assistants focus on supporting autonomy, independent living, and personal projects, and do not provide clinical or therapeutic interventions or assume clinical decision-making responsibilities. Integration within the organisation is facilitated through participation in service-specific meetings and meetings open to all staff members. There are no formal written protocols regulating the disclosure of lived experience; decisions regarding when and how to share personal experiences are addressed through ongoing accompaniment and reflection with the programme coordinator.

### 2.3. Study Setting and Participants

The study sample consisted of personal assistants with lived experience in mental health who were employed within the AFESMO’s peer support programme. The inclusion criteria required participants to have lived experience of mental health distress and to be formally employed as personal assistants within the framework of the program.

A purposive sampling strategy was applied based on these predefined inclusion criteria. The study sample consisted of personal assistants with lived experience of mental health distress who were employed within AFESMO’s peer support programme. At the time of the study, the team comprised eleven personal assistants; however, one did not meet the essential inclusion criterion of lived experience and was therefore excluded from participation. Consequently, the final sample included the totality of eligible personal assistants within the programme (*n* = 10). Participation was offered on a voluntary basis to all eligible individuals, without incentives or any consequences related to employment conditions. Table 1 presents their distribution by gender, age, prior relationship with the organisation, and length of time in the role.

### 2.4. Study Categories and Data Collection Tools

The development of the data collection tools was informed by the study objectives and a review of the relevant scientific literature. On this basis, an initial system of first- and second-level categories was established to guide the design of the interview and focus group guides. Based on these categories, semi-structured guides were developed for the individual interviews and the focus group. Their flexible use allowed the interviewers to adapt to the flow of each conversation and to address emerging issues, which subsequently contributed to the identification of new categories during the analysis. In addition, the guides included open-ended questions that encouraged participants’ free expression and enabled a deeper exploration of the topics they considered most significant. Table 2 presents the guiding questions used in both the individual interviews and the focus group:

### 2.5. Data Collection and Analysis

Fieldwork was conducted between September and October 2025. Participants were contacted through a reference person within the organisation, to facilitate access to the sample; however, participation was offered on a strictly voluntary basis and presented as independent of employment conditions or access to support services. Individual semi-structured interviews were conducted face-to-face in a safe and confidential setting within the organisation’s facilities, ensuring an atmosphere of trust and comfort. Interviews were carried out by the principal investigator (J.M.-O.), who, although a member of the organisation, had no supervisory, evaluative, or managerial role in relation to the participants and was affiliated with a different department. Participants were aware of the investigator’s organisational affiliation. All interviews were scheduled in advance with informed consent, and participants were informed about confidentiality procedures, data anonymisation, and who would have access to the data, which were used exclusively for research purposes. Interviews lasted approximately thirty minutes.

The qualitative analysis was conducted by a multidisciplinary research team with training in psychology and social sciences and prior experience in qualitative research. The individual interviews and the focus group were conducted by researchers with clinical and academic backgrounds in mental health and psychosocial care, while data analysis was carried out collaboratively by the research team. The team was aware of its positioning as health professionals and academics and adopted a reflexive stance throughout the analytical process, using analytic memos and systematic discussions to critically examine assumptions, emerging interpretations, and potential biases.

After the individual interviews were completed, a focus group was conducted with the same participants to contrast and enrich the individual accounts and to explore shared meanings related to professional experience. The session took place in a meeting room at the organisation’s facilities and was conducted in a trusting atmosphere, facilitated by participants’ prior familiarity with one another and with the moderator. The focus group lasted approximately two hours and was moderated by the principal investigator, with the support of an observer who recorded interactional processes and relevant non-verbal aspects.

All interviews, and the focus group were conducted in Spanish. Audio recordings were transcribed verbatim in Spanish. Quotations included in the manuscript were translated into English by members of the research team with bilingual proficiency. To ensure accuracy and conceptual equivalence, translations were reviewed collaboratively by the research team. The transcripts were anonymised using alphanumeric codes prior to analysis. and the resulting textual corpus was managed and analysed using MAXQDA software (version 24.11). Given the small size of the team, particular care was taken to remove identifying details, and access to the full dataset was restricted to the research team.

The analytical process followed a deductive–inductive and recursive qualitative content analysis approach [25,26,27]. As outlined previously, the analysis began with an initial system of predefined first- and second-level categories. During the coding process, additional first-level categories were created inductively from participants’ accounts. The coding framework was reviewed iteratively, returning to previously coded material to refine and consolidate the final analytical structure. Analytical memos were used throughout the process to document coding decisions and to support reflexive examination of how researchers’ organisational proximity and prior assumptions could influence data interpretation. Two researchers independently conducted the initial coding of a subset of transcripts, which served as the basis for developing a preliminary deductive codebook grounded in the study objectives and theoretical framework. The codebook was subsequently refined iteratively through the incorporation of emerging categories, the redefinition of codes, and the merging of conceptually overlapping categories, following a constant comparative approach. The remaining material was coded using the agreed-upon version of the codebook, with regular team meetings held to discuss the analytical progression.

Discrepancies in coding were resolved through discussion and consensus. For example, some excerpts were initially coded as “emotional distress” by one researcher and as “social isolation” by another; following joint review, these were integrated into a broader category of “psychosocial suffering,” encompassing both emotional and relational dimensions. When disagreements persisted, a third researcher was consulted to reach agreement.

To further enhance analytical rigour, a portion of the material was subjected to double coding. In addition, an audit trail was maintained, including analytic memos, successive versions of the codebook, and records of team discussions, ensuring transparency and traceability of the analytical process.

Conceptual saturation was assessed continuously during analysis and was considered achieved when successive interviews no longer yielded new codes or analytically relevant dimensions, and the categorical system demonstrated stability. Saturation was identified after the eighth individual interview; the two subsequent interviews confirmed thematic redundancy. The focus group conducted thereafter did not generate new categories but served to deepen, contrast, and refine the previously identified themes, reinforcing the coherence of the categorical system.

In addition, attention was paid to narratives that presented nuances or perspectives differing from the predominant pattern. These accounts were analysed in detail to adjust and enrich the analytical categories, rather than being discarded.

Table 3 summarises the final categorical system used in the analysis, integrating the initially defined first- and second-level categories with inductive categories identified as categories 6, 7, and 8.

To reinforce analytical rigour, triangulation among members of the research team was undertaken, including joint review of codes and systematic discussion of discrepancies until consensus was reached. The research team also maintained a reflexive stance throughout the process, critically examining how their own positioning and professional experience could influence data interpretation.

The reporting of this qualitative study was guided by the COREQ and SRQR reporting guidelines.

Analytical rigour was ensured through triangulation among members of the research team, joint review of the codes, and discussion of discrepancies until consensus was reached. The research team maintained a reflexive stance throughout the process, critically considering how their own positioning could influence the interpretation of the data.

### 2.6. Ethical Considerations

The study was conducted in accordance with the ethical principles of the Declaration of Helsinki. Informed consent was obtained from all participants, ensuring that they fully understood the conditions of confidentiality and their right to withdraw at any time. Measures were taken to protect participants’ emotional well-being, including the availability of support resources when needed. Furthermore, the research team maintained a reflexive and sensitive stance throughout the process. This included explicit consideration of potential power dynamics arising from organisational affiliation, anonymisation of data prior to analysis, and collective discussion of interpretations to reduce social desirability bias and enhance analytic credibility.

## 3. Results

Before presenting each thematic block, a quantitative synthesis of the coding process is provided, showing the frequency and proportion of coded segments associated with each category and theme within the data corpus. The percentage distributions reported in Table 4 are included for descriptive purposes only, to provide transparency regarding the coding process. Code frequency should not be interpreted as an indicator of analytical importance, as the interpretation of findings is grounded in the meaning and context of participants’ accounts. The most frequent code was ‘Professional identity and role clarity’ (24.49%). This highlights how often participants referred to this aspect of their work. ‘Perceived obstacles or barriers’ (15.42%) and ‘Perceived facilitators’ (15.19%) also appeared regularly and shaped much of the material analysed. While frequency alone does not determine analytical importance, these figures help situate the themes discussed in the following sections.

### 3.1. Professional Identity and Role Clarity: Balancing Lived Experience and Professionalisation

“Working from equal to equal” is perceived as the defining feature of the position, an approach grounded in empathy and understanding derived from lived experience. Lived experience thus becomes the specific value of the role, distinguishing it from the technical orientation of other professionals. Having gone through similar situations allows assistants to recognise difficulties, anticipate needs, and build trust from a place of equality and closeness:

“The people who can best help others going through mental health problems are precisely those who have ‘overcome’ them, in quotes, or have learned to live with them.”.

(Interview Participant 8, Pos. 44)

“[Personal assistance with peer support] is to show the users we support that it is possible, that there is hope.”

(Participant 5, Focus Group, Pos. 367)

The professional identity of personal assistants is therefore constructed between their own lived experience of mental health problems and the formalisation of their role as paid workers. This duality also generates certain doubts or tensions around the role, as reflected in the participants’ accounts:

“We also have the duality of fighting our own inner demons while at the same time helping other people overcome their illness.”.

(Focus Group Transcript, Pos. 415)

### 3.2. Work Environment: Tasks, Working Conditions, and Perceived Integration Within the Team

Within the broader construction of professional identity, several elements of the work environment contribute to shaping how assistants understand their role. Namely, the tasks they perform, the functions assigned to them, their working conditions, and their relationships with the rest of the team.

Clarity regarding job functions emerges as a key element in consolidating this hybrid identity, helping to define responsibilities and reduce role ambiguity:

“We were the first ones doing it at the time [referring to himself and another worker], and our functions were super clear.”.

(Focus Group Transcript, Pos. 63)

Regarding their assigned tasks and responsibilities, the work of personal assistants encompasses a wide variety of activities. In addition to direct support for service users; such as accompaniment, practical assistance, and emotional support; they are also given auxiliary tasks or asked to collaborate in other projects, such as helping with the Centre’s transport service, supporting group activities, or participating in a meal delivery service. These tasks, which are not strictly part of the personal assistant role, are experienced in different ways. Some view them as simple and manageable, even helpful for unplugging from direct support work, whereas others feel that such tasks do not align with what their role should entail, as reflected in the participants’ narratives:

“They hired me as a peer worker and suddenly things come up like, ‘go buy fabric softener,’ (…) I don’t know, things like that. And… that kind of thing doesn’t move anything in me. What I like is something that stirs me, and that doesn’t stir me at all.”.

(Interview Participant 2, Pos. 13)

Participants seem to agree that when too many tasks accumulate at once or when several cases must be managed simultaneously, a sense of disorder or overload appears. Some mention working more hours than planned or struggling to reconcile work with personal life:

“…sometimes I’ve felt fine, and other times I feel like… like a puppet. They call me many times, and I come for nothing. As if my time had no value. I don’t know how to explain it. Apart from this, I have my life and other things. And I’m the head of my household.”.

(Focus Group Transcript, Pos. 308)

Even so, personal assistants emphasise that the job offers considerable flexibility and is adapted to their personal needs. This helps them organize themselves better and gives them the sense that support is available when needed:

“I think they’re attentive to how we’re doing, to our difficulties, our limitations, our needs when it comes to working.”.

(Focus Group Transcript, Pos. 225)

Personal assistants describe generally feeling well supported by the rest of the team. They explain that they can ask questions, seek help, and express concerns without feeling judged. This sense of being listened to provides security and enables them to rely on other professionals when needed, as evidenced in the assistants’ accounts:

“When you have a doubt or something overwhelms you, being able to talk about it (with the coordinator) makes a big difference.”.

(Focus Group Transcript, Pos. 285)

These experiences were also reflected in concrete situations of interprofessional interaction, such as coordination with other professionals or access to supervision, where assistants described both opportunities for collaboration and ongoing uncertainties regarding role boundaries.

Some participants highlight the importance of case supervision and psychological support, while others emphasise that this need is not always fully met:

“It would be important to have a more regular supervision space, where you can talk about what affects you or about difficult cases. Because if not, you keep it to yourself and eventually it burns you out.”.

(Focus Group Transcript, Pos. 237)

Participants also note that, in some cases, prior coordination is insufficient. They sometimes begin accompanying a service user without having complete information or without clear agreement on who is responsible for each part of the process. This generates insecurity and makes it more difficult to decide how to intervene in certain situations:

“There are cases where it’s true that we go a bit blindly, but that’s because not even the professionals at AFESMO have more information.”

(Focus Group Transcript, Pos. 106)

Many assistants feel recognised and valued, although they also sense a barrier between themselves and other professionals, as the following excerpts show:

“There’s like this small barrier, as if we’re in no-man’s-land.”

(Focus Group Transcript, Pos. 296)

### 3.3. Disclosure of Lived Experience: Authenticity and Professional Discretion

Participants’ accounts reveal that disclosing one’s own lived experience is understood as a professional tool that strengthens trust and humanises the relationship, but its use requires prudence and sound judgement:

“I don’t start by saying, ‘I have the same thing as you’; I drop little hints depending on what I see.”.

(Individual Interview, Participant 1, Pos. 27)

“…I prefer not to say it at the beginning, because sometimes it also puts a label on you. And that’s not always helpful. Because we’re not only our illness, we’re a person with a set of characteristics, the illness being just one of them.”

(Interview Participant 3, Pos. 35)

### 3.4. Impact on Personal Assistants: Wellbeing, Purpose, and Emotional Vulnerability

Working as a personal assistant provides stability, routine, and a sense that the work is meaningful. Supporting others restores their confidence, strengthens their own recovery, and enhances their self-esteem. Witnessing the progress of the people they support makes them feel more secure, capable, and connected to life:

“I had never felt like waking up in the morning and actually wanting to go to work.”.

(Focus Group Transcript, Pos. 408)

However, the work also has a difficult side. Personal assistants experience emotional fatigue. When things go wrong, they may feel frustrated or guilty. Listening to difficult life stories stirs up their own experiences and requires extra effort to stay well, as the following accounts illustrate:

“But of course, if you’re not prepared and it catches you at a bad moment, it can bring up a lot, because you see so much of yourself reflected in the other person’s symptoms. It can stir you up emotionally.”.

(Interview Participant 7, Pos. 29)

### 3.5. Personal Assistants’ Perceptions of Impact on Service Users: Trust, Hope, and Autonomy

From the perspective of personal assistants, their work fosters trust among the people they support, grounded in shared experiences, and they perceived themselves as recovery role models who inspire hope. Participants described a shared goal of promoting trust and autonomy among programme beneficiaries:

“For me, it’s about showing users that yes, it’s possible-that there is hope.”.

(Focus Group Transcript, Pos. 367)

“For example, one of the girls I work with changed dramatically over the years. (…) Now she seems like a really happy girl. And I don’t know… it makes me really happy to see her like that now.”.

(Interview Participant 8, Pos. 36)

Despite these positive effects, personal assistants also identify certain limits or less favourable outcomes. At times, they explain, a degree of dependency may develop on the part of the person receiving support:

“The risk is (…) creating a strong emotional bond, (…) but we can’t be there every day. That’s the risk-that they become dependent on us.”.

(Interview Participant 9, Pos. 24)

These positive effects coexisted with the need to carefully manage relational boundaries, in order to support autonomy while avoiding the development of dependency.

### 3.6. Perceived Obstacles and Barriers: Emotional Strain, Blurred Boundaries, and Organisational Instability

Working as a personal assistant involves a high emotional load. Participants describe that constant exposure to others’ suffering and identification with service users’ stories can lead to psychological exhaustion, anxiety, or the reactivation of past experiences:

“…I was already getting too stressed. And I’m already in a delicate state of health. […] I can handle that workload for an hour, two hours, but maybe not three.”.

(Interview Participant 5, Pos. 13)

At the organisational level, assistants mention a lack of coordination and instability in scheduling and user assignments. The absence of information about each case’s goals or frequent changes in planning make follow-up difficult and generate insecurity:

“…we have to adapt, because that’s what this service is like [referring to constant changes in planning], and we end up working on the fly; I think that could be improved.”.

(Focus Group Transcript, Pos. 229)

### 3.7. Perceived Facilitators: Institutional Support, Care, and Recognition

Personal assistants highlight several factors that facilitate their work and help prevent burnout. Some have already been mentioned, such as support from project coordination, supervision and access to psychological support, flexible working conditions and professional recognition, as the following narratives illustrate:

“…I feel recognised, valued, and well supported.”.

(Focus Group Transcript, Pos. 294)

In addition to these, informal support among colleagues and the possibility of sharing difficulties with coordinators are also perceived as helpful elements in their day-to-day work:

“…you always have a coordinator there who can help you. I think they respond very quickly in that regard, which I see as very positive.”.

(Focus Group Transcript, Pos. 33)

“…lunchtime is great because you get together with colleagues […] and I think that really helps us.”.

(Focus Group Transcript, Pos. 353)

### 3.8. Suggestions for Improvement: Organisation, Communication, and Training

Personal assistants propose several improvements to strengthen their work and enhance the quality of support provided. They emphasise the need for better internal communication and more information about each case before beginning to work with a person:

“…it would be good to at least focus on each user’s goals-what they want to achieve-in order to help address their difficulties in life.”.

(Focus Group Transcript, Pos. 176)

They also highlight the importance of having group spaces for coordination and supervision, where they can share doubts, review situations, and ensure their voices are heard:

“…that our voices be heard […] that they hold this kind of meeting to share information, to discuss, to see how we can continue improving.”.

(Focus Group Transcript, Pos. 414)

Finally, they express the need for more specific training to face certain situations and for a dedicated office space within the organisation:

“…to receive specific training from a professional here on the cases we’ve raised […] trying to find solutions together.”.

(Focus Group Transcript, Pos. 234)

“We are missing our own physical space, for example one that says *Personal Assistants*. That would mean having a place for us within the organisation, and right now we don’t have it.”.

(Focus Group Transcript, Pos. 303)

### 3.9. Inductive Codes: Families, Networks, and Motivations

Among the unanticipated themes, several cross-cutting aspects emerged, such as the role of families -who may facilitate the process by supporting the intervention, while others hinder it through overprotective attitudes or a lack of collaboration:

“Families often do a lot of harm to a person with a mental illness (…) but not always. I’ve also seen cases where it’s quite the opposite.”.

(Focus Group Transcript, Pos. 215)

Another relevant aspect is the support network among personal assistants themselves, which functions as a spontaneous space for venting, exchange, and mutual support, as evidenced in their testimonies below:

“Something we’ve talked about several times is what we used to do before meetings, and that we’ve now lost… Getting that back would be helpful. It was like: ‘Hey, how are you doing with this?’ (…) That way, we could help each other more easily.”.

(Focus Group Transcript, Pos. 234)

Finally, many participants highlight personal motivation as a key factor: working as personal assistants allows them to feel useful, to grow, and to maintain a clear sense of purpose, which strengthens their commitment and consistency:

“…when I saw that I could help people who had gone through things similar to mine […] I felt like I would be doing a bit of good in the world.”.

(Interview Participant 8, Pos. 4)

These inductive themes complement the main analytical categories by highlighting the relational and motivational contexts that shape the everyday practice of peer-based personal assistance.

## 4. Discussion

The findings of this study offer a coherent picture of how the personal assistance framework shapes everyday practice, team integration, and the meanings that participants attribute to their role. The combination of structured tasks, organisational support, and the intentional use of lived experience appears to generate both opportunities and tensions that resonate with previous literature on peer support and personal assistance. These elements provide a foundation for interpreting how participants negotiate their place within services, how they understand their contribution, and how they navigate the boundaries of a role situated between personal history and formal professional expectations. It is important to note that the findings presented in this section reflect personal assistants’ interpretations and experiences, rather than independently assessed organisational processes or service user outcomes.

The professional identity of personal assistants with lived experience is hybrid: it moves between personal experience and professionalisation. This “liminal position” [6], associated with role ambiguity and a lack of clear definition, aligns with previous research on the integration of peer support workers into mental health services [11]. At our organisation, embedding peer support within the formal contractual framework of Personal Assistance, as recognised in Law 39/2006, appears to reduce ambiguity and strengthen the legitimacy of the role.

Integrating peer support within the structure of Personal Assistance contributes to a clearer definition of tasks and functions, creating a recognised position within the organisation and reducing role ambiguity. While previous studies on the professionalisation of peer support consistently describe liminality and role ambiguity as persistent challenges—often linked to unclear employment conditions and peripheral organisational positioning—the findings of this study suggest that embedding peer support within a formally regulated personal assistance framework may partially mitigate these tensions. In contrast to others peer support models described in the literature [5,7], the Personal Assistance framework provides contractual stability, clearer task delineation, and institutional recognition, which appear to reshape how liminality is experienced and negotiated in everyday practice. However, tensions persist between the technical and experiential dimensions of the role: assistants continue to situate themselves “between two worlds,” neither fully professionals nor service users [28]. Some of this residual ambiguity arises from the assignment of auxiliary or cross-departmental tasks that extend beyond direct peer support activities, generating doubts about role boundaries. Consistent with previous research, these findings underscore that, even within formal frameworks, professional boundaries are continually negotiated in practice and remain highly dependent on the degree of interprofessional coordination [29,30]. From an organisational and implementation perspective, these experiences can be understood as emerging not only from individual biographies, but also from how peer roles are formally embedded, supported, and regulated within service structures, shaping expectations, boundaries, and legitimacy in everyday practice.

Our data add a further nuance likely linked to the use of Personal Assistance as a framework for peer support: self-disclosure of lived experience is not spontaneous or automatic. Instead, peer professionals disclose their mental health experience selectively and deliberately. Consistent with other authors [9,31,32], self-disclosure functions as a professional tool used strategically, according to the needs of each case, to build rapport and guide the support relationship, while avoiding excessive public exposure of personal experience [16]. Evidently, the management of experiential knowledge aligns with the recovery literature: lived experience provides practical insight that enhances understanding of situations, increases empathy, and sustains hope [3,33]. Nevertheless, its legitimacy does not rest solely on explicit recognition; it must be integrated into work processes and decision-making structures [9].

The integration of peer support workers remains a challenge. The lack of formal recognition, role ambiguity, and certain ambivalent attitudes can lead to peripheral or symbolic positions (tokenism) [14]. It is well established that integration entails organisational challenges such as the development of more consistent structures for support, supervision, and coordination [3], institutional recognition [34], and mandatory training for all staff on the peer role [11]. Within the Spanish context, Eiroa-Orosa and Sánchez-Moscona [16] also highlight the absence of a stable legal and labour framework as a barrier to implementation.

The Personal Assistance framework provides clarity and security through formal contracts, a defined set of tasks and functions, and structured expectations, reducing uncertainty and legitimising the role in the eyes of staff, service users, and families. The shortcomings in initial information-sharing (as highlighted in the focus group) point to the need to formalise information flows and address the question of what information should be shared within the team and what should not. In our study, assistants perceive themselves as occupying an intermediate position between service users and technical staff. Even so, they note a strong institutional effort to support them and avoid leaving them isolated, which enhances their sense of belonging. Close coordination acts as a bridge between organisational levels and translates into a feeling of being “looked after” and having support available when needed. Evidence shows that the absence of clear policies for supervision and coordination constitutes a barrier to the consolidation of such programmes [16].

The findings also underscore the importance of informal support networks among personal assistants themselves. This everyday (non-formalised) support operates as a safe space for emotional release, sharing doubts, and coping with emotional strain, consistent with the literature on the value of peer community in preventing burnout and isolation. In the same vein, recent studies recommend combining supervision with mentoring, buddy systems, and peer-support networks to reduce isolation and prevent emotional overload [9,30]. However, communication gaps with traditional professional roles persist, limiting mutual understanding. Without formal communication mechanisms and participatory leadership, integration depends too heavily on goodwill [10]. The key lies in moving from symbolic recognition to structural participation: co-governance, a voice in planning and evaluation, and real redistribution of power [14]. In this regard, demands such as having a dedicated physical space for personal assistants reflect the need to materialise professional recognition and strengthen their identity within the organisation.

Peer work combines a high emotional load with significant potential for growth. It brings meaning and pride, but it can also be draining. The evidence is clear: experiential proximity enhances empathy and hope [2,3] while the sustained emotional demands of peer work, particularly in the absence of adequate support, increase the risk of compassion fatigue [29]. This risk decreases when there are psychological safety and a climate that legitimises discussing difficulties and seeking help, both associated with better performance. Operationally, clear job descriptions, sensitive labour policies, and ongoing support help maintain boundaries and protect workers [9]. Our findings confirm the value of care structures (psychological supervision and accessible coordination) as protective factors. Emotional management is learned and exercised as a professional competency. Clear protocols, defined functions, and continuous training help safeguard boundaries and prevent over-involvement. Nonetheless, part of the emotional strain is managed informally or in isolation, creating a risk that it remains hidden underscoring the continued need for structured supervision and ongoing support systems.

While these findings primarily reflect individual lived experiences, they also point to broader organisational implications, particularly regarding workload distribution, supervision arrangements, and the need for clear role boundaries to ensure sustainable peer-based support.

The results also show that the role of service users’ families can function either as a facilitator or as a barrier. Overprotective attitudes or a lack of understanding of the personal assistant’s role may hinder progress and generate tensions that require coordination and ongoing education. Complementarily, the positive impact on service users—increased hope, trust, and autonomy—is consistent with the well-established evidence on the benefits of peer support [4], reinforcing its value within the Personal Assistance framework.

These interpretations should be considered in light of the exploratory nature of the study, which was conducted in a single organisational context with a small sample, and therefore aim to inform understanding rather than to support generalisable conclusions.

### 4.1. Limitations of the Study

As data were collected exclusively from personal assistants, references to team integration, organisational functioning, and impact on service users should be understood as perceived effects reported by participants, rather than as independently assessed outcomes. The design is cross-sectional and based on self-reported accounts (interviews and focus group), without temporal follow-up or observational data of work in context; moreover, no quantitative indicators of user outcomes or organisational metrics (e.g., retention, absenteeism, sick leave) were included. Addressing these gaps would require multicentre and comparative studies, longitudinal designs to track the evolution of the role and performance, mixed methods approach (qualitative and quantitative), or research that incorporates the perspectives of service users, other professional profiles, and families. There are also aspects highlighted in the previous literature that do not appear in our data, for example, employment conditions such as remuneration or job stability, which could be explored in future studies.

In addition, the recruitment procedures and the positionality of the research team may have influenced the data. Participants were recruited within a single organisation, and data collection was conducted by a researcher who belongs to that organisation, which may have shaped how experiences were narrated, including a tendency towards social desirability or a greater emphasis on positive aspects. Although several measures were adopted to minimise these effects—such as voluntary participation, the absence of hierarchical or supervisory relationships, and assurances of confidentiality and data anonymisation—these factors should be considered when interpreting the findings.

### 4.2. Relevance for Practice and Future Research

This study highlights several implications for practice within mental health services. Clear contractual and functional frameworks strengthen the professional identity and legitimacy of peer support workers, facilitating their integration within multidisciplinary teams. Equally important are organisational cultures that prioritise supervision, accessible coordination, and emotional safety, as these elements help prevent emotional exhaustion and support the long-term sustainability of experiential roles. Providing dedicated spaces, improving internal communication channels, and ensuring structured participation in decision-making processes further contribute to reducing feelings of marginalisation and consolidating the role of Personal Assistants. Recognising experiential knowledge as a legitimate professional asset requires moving from symbolic inclusion to structural participation, ensuring that the voices of staff with lived experiences have a meaningful impact on service planning, evaluation, and development.

Future research could build on these findings through multicentre or comparative designs that explore how different organisational contexts shape the development of peer-based Personal Assistance models. Longitudinal approaches would allow examination of how professional identity, role boundaries, and emotional wellbeing evolve over time. Mixed-methods studies could triangulate qualitative insights with indicators such as user outcomes, job satisfaction, turnover, or absenteeism. Incorporating the perspectives of service users, families, and other professionals would also offer a more comprehensive understanding of relational dynamics, organisational integration, and the perceived value of experiential knowledge. Finally, further research is needed to explore how training, accreditation pathways, and competency frameworks contribute to the consolidation and long-term sustainability of peer-support roles within Personal Assistance programmes.

## 5. Conclusions

The personal assistance model represents an innovative experience within the Spanish context, integrating peer support into the formally recognized role of the Personal Assistant. According to participants accounts, this approach provides contractual stability and functional clarity, which were perceived as helping to reduce the ambiguity traditionally associated with peer support roles. The analysis identifies three central pillars of the model: (a) a clear and replicable contractual structure; (b) a well-defined framework of tasks and functions that strengthens professional recognition among technical staff, families, and service users; and (c) a culture of care grounded in ongoing supervision and emotional support.

Overall, the findings suggest that, from the perspective of personal assistants, Personal Assistance may constitute a viable pathway for the professionalisation of peer support, while recognising the exploratory nature and contextual limits of the study. The role combines purpose, experiential knowledge, and practical competencies which participants perceived as fostering trust, hope, and autonomy among service users while also supporting the assistants’ own recovery and sense of professional identity. Nonetheless, important challenges remain, such as information gaps, irregular communication with traditional professional roles, and a partial sense of belonging, the resolution of which will be essential to advance towards a truly integrative model that values experiential knowledge without diluting its specificity.

## Figures and Tables

**Table 1:** Sociodemographic characteristics of personal assistants with lived experience in mental health.

Participant ID	Gender	Age (Years)	Previous Relationship with the Organisation	Length of Service in the Role (Months)
Participant 1	Male	47	Yes	17
Participant 2	Female	57	Yes	52
Participant 3	Female	42	Yes	15
Participant 4	Male	32	Yes	39
Participant 5	Male	43	Yes	41
Participant 6	Male	39	No	11
Participant 7	Male	30	No	7
Participant 8	Male	21	Yes	31
Participant 9	Female	54	No	8
Participant 10	Male	58	Yes	12

**Table 2:** Guiding questions for individual interviews and focus group.

Individual Interview	Focus Group
How would you describe, from your own experience, what it means to be a personal assistant providing peer support?	What factors have facilitated the development of your work?
What types of situations or tasks are part of your daily activities? Which are the easiest or most difficult to manage?	What difficulties or barriers have you encountered?
Do you think your work differs depending on whether the people you support know or don’t know that you have personal experience with mental health issues?	Do you feel that you are part of the care system, or are there moments when you feel on the margins?
What effects (positive or negative) do you think your work has on the people you support?	What do you consider most valuable in the peer-support personal assistance programme?
What effects (positive or negative) can this work have on your own recovery process?	What things would you change or improve if you could?

**Table 3:** Final structure of the study’s categorical system.

First-Level Categories	Second-Level Categories
Deductive	
1. Professional identity and role clarity	1.1. Working conditions 1.2. Assigned tasks 1.3. Team integration
2. Impact on the personal assistant	2.1. Negative effects on the personal assistant 2.2. Positive effects on the personal assistant
3. Impact on service users	3.1. Negative effects on service users 3.2. Positive effects on service users
4. Disclosure of lived experience of mental health	-
5. Barriers and facilitators in role performance	5.1. Perceived facilitators 5.2. Suggestions for improvement
Inductive	
6. Influence of service users’ families	-
7. Informal support networks within the organisation	-
8. Motivations	-

**Table 4:** Number of coded segments in each code.

Code	Segments	%
Professional Identity and Role Clarity	108	24.49%
Barriers in role performance	68	15.42%
Facilitators in role performance	67	15.19%
Impact on the Personal Assistants	49	11.11%
Impact on Service Users	44	9.98%
Suggestions for Improvement	38	8.62%
Inductive Codes	34	7.71%
Disclosure of lived experience of mental health	33	7.48%
TOTAL	441	100.00

## Data Availability

The data presented in this study are not available due to privacy.

## References

[1] Mion A.B.Z., Arena Ventura C.A. (2024). The WHO QualityRights Initiative and its use worldwide: A literature review. Int. J. Soc. Psychiatry.

[2] Davidson L., Bellamy C., Guy K., Miller R. (2012). Peer support among persons with severe mental illnesses: A review of evidence and experience. World Psychiatry.

[3] Gillard S., Foster R., White S., Barlow S., Bhattacharya R., Binfield P., Eborall R., Faulkner A., Gibson S., Goldsmith L.P. (2022). The impact of working as a peer worker in mental health services: A longitudinal mixed methods study. BMC Psychiatry.

[4] Smit D., Miguel C., Vrijsen J.N., Groeneweg B., Spijker J., Cuijpers P. (2022). The effectiveness of peer support for individuals with mental illness: Systematic review and meta-analysis. Psychol. Med..

[5] Gates L.B., Akabas S.H. (2007). Developing strategies to integrate peer providers into the staff of mental health agencies. Adm. Policy Ment. Health.

[6] Simpson A., Oster C., Muir-Cochrane E. (2018). Liminality in the occupational identity of mental health peer support workers: A qualitative study. Int. J. Ment. Health Nurs..

[7] Jones N., Niu G., Thomas M., Riano N.S., Hinshaw S.P., Mangurian C. (2019). Peer specialists in community mental health: Ongoing challenges of inclusion. Psychiatr. Serv..

[8] Tanguy L., Dubar C. (1993). La socialisation. Construction des identités sociales et professionnelles. Rev. Fr. Sociol..

[9] Harris A., Stanczyk A., McCallum D. (2025). Hiring and Supporting Peer Support Staff: Strategies from Three NextGen Employment Programs.

[10] Reeves V., McIntyre H., Loughhead M., Halpin M.A., Procter N. (2024). Actions Targeting the Integration of Peer Workforces in Mental Health Organisations: A Mixed-Methods Systematic Review. BMC Psychiatry.

[11] Robertson S., Leigh-Phippard H., Robertson D., Thomson A., Casey J., Walsh L.J. (2024). What Supports the Emotional Well-Being of Peer Workers in an NHS Mental Health Service?. Ment. Health Soc. Incl..

[12] Prat Vigué G., Cano Prieto I., del Río Sáez R., Vilanova Masana R., Simó Algado S. (2022). Training Peer Support Workers in Mental Health Care: A Mixed Methods Study in Central Catalonia. Front. Psychiatry.

[13] Zabaleta-González R., Lezcano-Barbero F., Martínez-Pérez A., Casado-Muñoz R. (2023). Métodos e instrumentos de evaluación en los programas de formación de pares para personas con problemas de salud mental: Revisión documental. Interface-Comun. Saúde Educ..

[14] Pollice G., Bodini C.F., Menchetti M., Da Mosto D., Negrogno L., Betti L., Furlan M., Quaranta I. (2025). “Is the Service Ready?”: Integrating Peer Support Workers Within Community Mental Health: An Ethnographic Study from Trieste and Its Region. Community Ment. Health J..

[15] Holm M.E., Jørgensen K., Andreasson K., Vinberg M., Nordentoft M., Midtgaard J. (2025). ‘I’ve Seen You at Your Worst, but I Still Want to Be Your Friend’: A Qualitative Study of Peer Support Exchange from the Perspective of Participants in Online Communities Centred around Self-Harming and Suicidal Behaviour. Digit. Health.

[16] Eiroa-Orosa F.J., Sánchez-Moscona C. (2023). Implementación de la figura de agente de apoyo entre iguales en salud mental: Una perspectiva internacional en el contexto de su implementación en Cataluña. Salud Colect..

[17] IMSERSO (2024). Informe Estadísticos del Sistema para la Autonomía y Atención a la Dependencia. https://imserso.es/el-imserso/documentacion/estadisticas/sistema-autonomia-atencion-dependencia-saad/estadisticas-mensual.

[18] Lozano de las Morenas Á., Robles Peña E., Quezada M.Y. (2019). Asistencia Personal en Salud Mental. Experiencia de Salud Mental en Castilla y León. http://riberdis.cedid.es/handle/11181/5983.

[19] Cabanes Aracil M., Iantzi Vicente S., Pereñíguez Olmo M.D., Morales Ortiz J., Mesas Fernández J. (2024). La asistencia personal y el apoyo de pares en Salud Mental: Una revisión sistemática. Innovación en la Educación y Práctica Profesional en Salud: Estrategias Transformadoras.

[20] Gehrig R., Palacios Ramírez J. (2014). Guía de Criterios Básicos de Calidad en la Investigación Cualitativa.

[21] Taylor S.J., Bogdan R. (1992). Introducción a los Métodos Cualitativos de Investigación.

[22] Valles M.S. (2002). Entrevistas Cualitativas. Cuadernos Metodológicos.

[23] Rodas Pacheco F.D., Salazar V.G.P. (2020). Grupos Focales: Marco de Referencia para su Implementación. INNOVA Res. J..

[24] Merton R.K. (1987). The Focussed Interview and Focus Groups: Continuities and Discontinuities. Public Opin. Q..

[25] Elo S., Kyngäs H. (2008). The Qualitative Content Analysis Process. J. Adv. Nurs..

[26] Assarroudi A., Heshmati Nabavi F., Armat M.R., Ebadi A., Vaismoradi M. (2018). Directed qualitative content analysis: The description and elaboration of its underpinning methods and data analysis process. J. Res. Nurs..

[27] Fereday J., Muir-Cochrane E. (2006). Demonstrating Rigor Using Thematic Analysis: A Hybrid Approach of Inductive and Deductive Coding and Theme Development. Int. J. Qual. Methods.

[28] Vandewalle J., Debyser B., Beeckman D., Vandecasteele T., Van Hecke A., Verhaeghe S. (2016). Peer Workers’ Perceptions and Experiences of Barriers to Implementation of Peer Worker Roles in Mental Health Services: A Literature Review. Int. J. Nurs. Stud..

[29] Reeves V., Loughhead M., Halpin M.A., Procter N. (2024). “Do I Feel Safe Here?” Organisational Climate and Mental Health Peer Worker Experience. BMC Health Serv. Res..

[30] Yiu E.K.L., Wong S.M.Y., Lee J.K.Q., Ng Z.L.Y., Leung D.K.Y., Chan W.C., Wong G.H.Y., Lum T.Y.S. (2025). Navigating Challenges in Peer Support Work: Perspectives of Peer Supporters from a Stepped Care Intervention for Older Adults with Depressive Symptoms. Health Expect..

[31] Ibrahim N., Thompson D., Nixdorf R., Kalha J., Mpango R., Moran G., Mueller-Stierlin A., Ryan G., Mahlke C., Shamba D. (2020). A Systematic Review of Influences on Implementation of Peer Support Work for Adults with Mental Health Problems. Soc. Psychiatry Psychiatr. Epidemiol..

[32] Ramesh M., Charles A., Grayzman A., Hiltensperger R., Kalha J., Kulkarni A., Mahlke C., Moran G.S., Mpango R., Mueller-Stierlin A.S. (2023). Societal and Organisational Influences on Implementation of Mental Health Peer Support Work in Low-Income and High-Income Settings: A Qualitative Focus Group Study. BMJ Open.

[33] Davidson L., O’Connell M., Tondora J., Styron T., Kangas K. (2006). The Top Ten Concerns About Recovery Encountered in Mental Health System Transformation. Psychiatr. Serv..

[34] Saad G., Honey A., Schaecken P., Scanlan J.N. (2024). Strategies and Supports Used by Mental Health Peer Workers to Facilitate Role Performance and Satisfaction. Adv. Ment. Health.

